# A global meta‐analysis reveals higher variation in breeding phenology in urban birds than in their non‐urban neighbours

**DOI:** 10.1111/ele.14099

**Published:** 2022-09-22

**Authors:** Pablo Capilla‐Lasheras, Megan J. Thompson, Alfredo Sánchez‐Tójar, Yacob Haddou, Claire J. Branston, Denis Réale, Anne Charmantier, Davide M. Dominoni

**Affiliations:** ^1^ School of Biodiversity, One Health and Veterinary Medicine University of Glasgow Glasgow UK; ^2^ Département des Sciences Biologiques Université du Québec à Montréal Montreal Canada; ^3^ Centre d'Ecologie Fonctionnelle et Evolutive Université de Montpellier, CNRS, EPHE, IRD Montpellier France; ^4^ Department of Evolutionary Biology Bielefeld University Bielefeld Germany

**Keywords:** global change, habitat heterogeneity, life‐history traits, meta‐analysis, phenotypic variation, synthesis, urban ecology, urbanisation

## Abstract

Cities pose a major ecological challenge for wildlife worldwide. Phenotypic variation, which can result from underlying genetic variation or plasticity, is an important metric to understand eco‐evolutionary responses to environmental change. Recent work suggests that urban populations might have higher levels of phenotypic variation than non‐urban counterparts. This prediction, however, has never been tested across species nor over a broad geographical range. Here, we conducted a meta‐analysis of the avian literature to compare urban versus non‐urban means and variation in phenology (i.e. lay date) and reproductive effort (i.e. clutch size, number of fledglings). First, we show that urban populations reproduce earlier and have smaller broods than non‐urban conspecifics. Second, we show that urban populations have higher phenotypic variation in laying date than non‐urban populations. This result arises from differences between populations within breeding seasons, conceivably due to higher landscape heterogeneity in urban habitats. These findings reveal a novel effect of urbanisation on animal life histories with potential implications for species adaptation to urban environments (which will require further investigation). The higher variation in phenology in birds subjected to urban disturbance could result from plastic responses to a heterogeneous environment, or from higher genetic variation in phenology, possibly linked to higher evolutionary potential.

## Graphical abstract

Phenotypic variation is an important metric to understand eco‐evolutionary responses to environmental change. We conduct a meta‐analysis of the bird literature to compare urban versus non‐urban means and variation in phenology and reproductive effort. We show that urban populations reproduce earlier, have smaller broods and have higher phenotypic variation in laying date than non‐urban conspecifics.

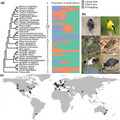

## INTRODUCTION

Humans have drastically changed environmental conditions on Earth, particularly since the invention of agriculture during the Neolithic Revolution. The footprint of human activity is most pronounced in urban environments, where microclimatic conditions, biogeochemical cycles and sensory landscapes are considerably different from those in non‐urban habitats (Grimm et al., 2008). Perhaps not surprisingly, multiple shifts in animal and plant phenotypes have been associated with the novel conditions and selective pressures found in cities (Hendry et al., 2017). Indeed, numerous studies have reported divergent phenotypes between urban and non‐urban populations in phenological, morphological, behavioural and reproductive traits (e.g. Alberti et al., 2017; Diamond et al., 2018; Campbell‐Staton et al., 2020; reviewed in Johnson & Munshi‐South, 2017; Lambert et al., 2020; Diamond & Martin, 2021). Most studies in urban ecology and evolution to date have focused on urban effects on *mean* phenotypes, and no study has explicitly investigated how urbanisation affects phenotypic *variation*. The extent to which populations can adapt to urban environments could be partly associated with how urbanisation affects their phenotypic variation (Thompson et al., 2022). Phenotypic variation is tightly linked to eco‐evolutionary processes (Fusco, 2001; Pavlicev et al., 2011): it is an essential condition for current selection, it results from past selection pressures, and it depends on gene flow and phenotypic plasticity. As such, assessing how urbanisation affects phenotypic variation can help us understand the potential for future phenotypic changes in urban environments and the eco‐evolutionary implications of such changes (Thompson et al., 2022).

Recent single‐species studies suggest that phenotypic variation could be affected by urbanisation (Caizergues et al., 2018; Gorton et al., 2018; Thompson et al., 2022). For example in species with limited dispersal ability (i.e. whose dispersal occurs at a smaller scale than the scale at which the urban habitat varies), adaptation to local conditions could increase phenotypic variation within the urban matrix in heterogeneous urban environments. Findings from urban and non‐urban meta‐populations of the common ragweed (*Ambrosia artemisiifolia*) are consistent with this prediction as inter‐population variation in several fitness proxies was greater in urban compared to non‐urban environments (Gorton et al., 2018). A meta‐analysis of selection strength found weaker selection occurring in human‐disturbed populations (Fugère & Hendry, 2018; note that this analysis did not specifically test the effect of urbanisation on selection strength and only included one study directly associated with urbanisation), which if extrapolated to the urban context, could lead to higher phenotypic variation in urban populations compared to their non‐urban counterparts. Overall, these studies converge with the notion that urban populations could display higher levels of phenotypic variation due to several eco‐evolutionary processes. These findings also highlight that the extent to which urbanisation might impact phenotypic variation likely depends on the interplay between the temporal and spatial scale at which environmental conditions fluctuate in the urban habitat, as well as on the species' longevity and dispersal ability (Thompson et al., 2022).

The temporal scale at which differences in phenotypic variation between urban and non‐urban habitats manifest can help us evaluate their ecological causes, and is likely to determine the eco‐evolutionary implications of increased phenotypic variation in urban habitats (Thompson et al., 2022). First, urban populations could display higher phenotypic variation than non‐urban populations within a given breeding season (i.e. intra‐annual variation; as a result, for example of consistent differences in landscape heterogeneity between habitats; Pickett et al., 2017). Second, urban populations could display higher phenotypic variation than non‐urban populations due to larger yearly fluctuations in environmental conditions (i.e. inter‐annual variation; if, e.g. urban populations are more sensitive to changes in weather conditions), with or without intra‐annual differences in phenotypic variation between urban and non‐urban populations. In the latter scenario, similar levels of phenotypic variation would be exposed to natural selection in short‐lived species (e.g. annual species).

Urban environments have been referred to as spatially more heterogeneous than non‐urban habitats of the same geographical area (Pickett et al., 2017). High urban habitat heterogeneity could increase phenotypic variation compared to adjacent non‐urban habitats if, for example urban organisms change their phenotype according to local environmental conditions (e.g. through either developmental or later‐life phenotypic plasticity). The empirical assessment of this idea, however, largely depends on the scale at which urban habitat heterogeneity is measured, the spatial scale at which the organism of interest operates and the heterogeneity of the non‐urban habitat of reference (Pickett et al., 2017; Uchida et al., 2021). For example a megacity could be spatially heterogeneous, containing a diverse array of habitats (e.g. multiple urban parks with different ecological conditions, a varying level of impervious surface, etc.), and, thus, be overall vastly more heterogeneous than a neighbouring non‐urban habitat. However, species could reduce the range of environmental conditions that they experience through matching habitat choice (e.g. Muñoz et al., 2014), limiting the potential effect of urban habitat heterogeneity on phenotypic variation. Therefore, measuring habitat heterogeneity at different spatial scales will be paramount to understand the potential association between habitat heterogeneity and increased phenotypic variation in urban areas.

**FIGURE 1:**
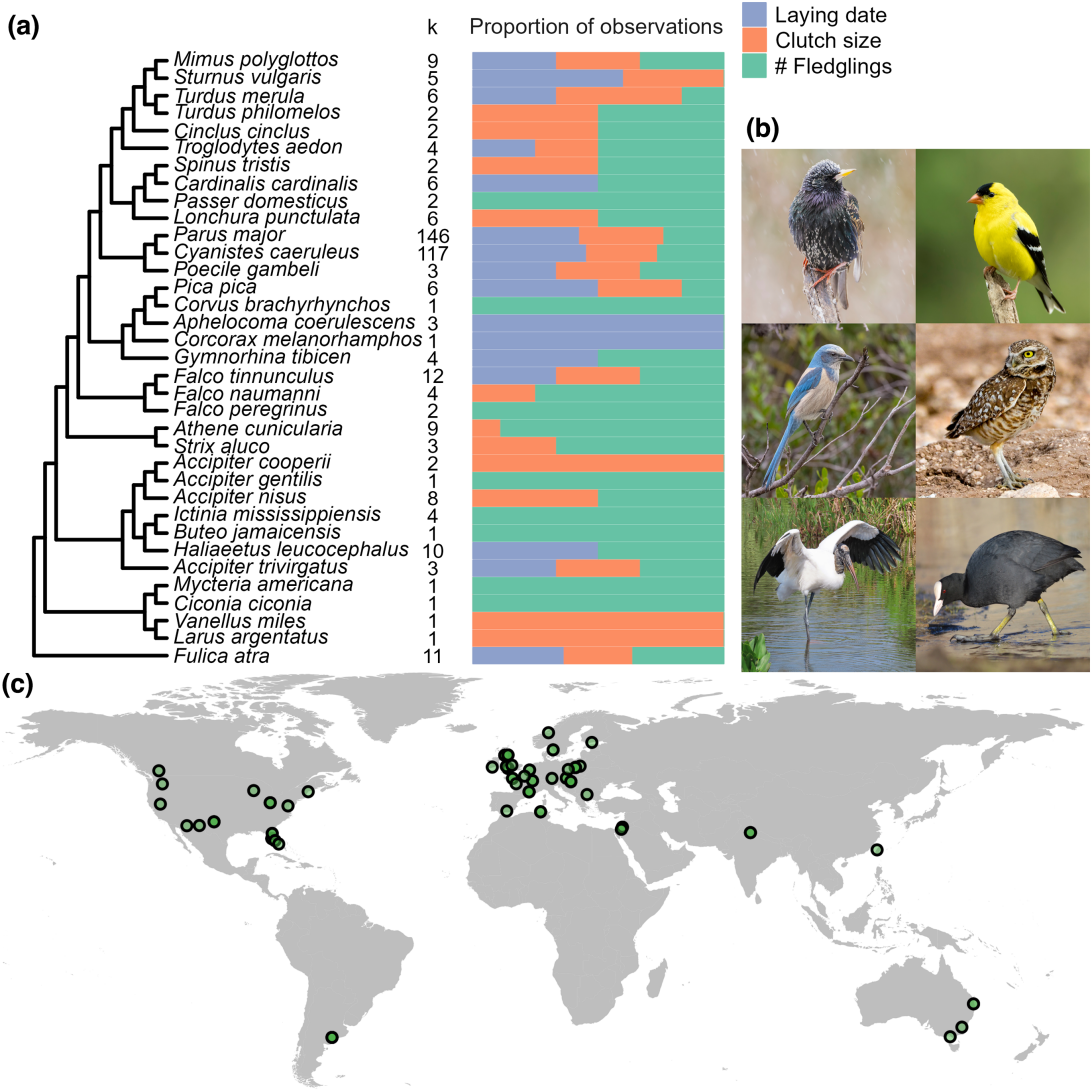
Phylogenetic and geographical breadth of the meta‐analytic data. (a) Phylogenetic tree of the 35 avian species included in the meta‐analysis along with the number of effect sizes (i.e. urban–non‐urban comparisons) included per species (‘*k*’; which may encompass multiple years of study from the same publication) and the proportion of observations for each life‐history trait (purple: Laying date; orange: Clutch size; Green: Number of fledglings). (b) Our meta‐analysis included a broad range of species, as examples, left to right from top to bottom: *Sturnus vulgaris*, *Spinus tristis*, *Aphelocoma coerulescens*, *Athene cunicularia*, *Mycteria americana* and *Fulica atra*. All images are copyright free (CC—Public Domain Mark 1.0. Authors: Shenandoah National Park [first two images], Mike Carlo/U.S. Fish & Wildlife Service, Jennifer Soos, Susan Young and Ekaterina Chernetsova) and were extracted from www.flickr.com. (c) Global map (excluding Antarctica) showing the location of each study included in the meta‐analysis. Each point represents one study area in which one or more urban–non‐urban pairs of populations were sampled across a varying number of years.

Here, we investigate how urbanisation impacts mean phenotypic values and phenotypic variation using a meta‐analysis of 399 paired urban and non‐urban comparisons of avian life‐history traits (laying date, clutch size and number of fledglings) published between 1958 and 2020 including 35 bird species (Figure 1). We use paired within species urban–non‐urban comparisons to investigate the following questions: (i) Is urbanisation associated with shifts in mean life‐history traits? (ii) Is urbanisation associated with changes in variation in life‐history traits? (iii) What is the temporal and spatial scale at which urbanisation correlates with changes in phenotypic variation? Based on previous research (Chamberlain et al., 2009; Sepp et al., 2018), we predict that urban bird populations display on average earlier phenology, smaller clutch size and lower number of fledglings than non‐urban populations. We also predict increased phenotypic variation in urban populations compared to non‐urban populations for all three traits examined (see above). We disentangle urban effects on phenotypic variation across different temporal and spatial scales, suggesting an ecological mechanism for the effects of urbanisation on avian phenotypic variation. This study provides, for the first time, meta‐analytical evidence that urban conditions can magnify phenotypic variation in phenology and highlights the potential role of increased habitat heterogeneity in urban areas as an ecological mechanism underlying this effect.

## MATERIAL AND METHODS

### Literature review

We began our literature search by inspecting two published reviews on the impact of urbanisation on avian biology (Chamberlain et al., 2009; Sepp et al., 2018). As we were interested in how phenology and reproduction were affected by urbanisation, we identified studies cited in Chamberlain et al. (2009) (*n* = 37) and Sepp et al. (2018) (*n* = 32) that could contain either raw data, or mean and variance estimates for first clutch laying initiation (hereafter laying date), clutch size and number of nestlings fledged per breeding attempt (hereafter number of fledglings), for paired urban and non‐urban populations (see details below). Then, we performed four searches of the Web of Science Core Collection on the 27th of October 2020 (databases covered: SCI‐EXPANDED—1900–present, SSCI—1956–present, A&HCI—1975–present, BKCI‐S—2005–present, BKCI‐SSH—2005–present and ESCI—2015–present) to recover studies published since 1900 and including all languages and all document types. We performed the following four searches on the Web of Science Core Collection: (1) TS = (“urban*” AND (“bird*” OR “aves” OR “avian” OR “ornithol*” OR “passerine*” OR “passeriform*” OR “songbird*” OR *list of bird genera*) AND (“laying date” OR “lay date” OR “first egg” OR “clutch size” OR “eggs laid” OR “number of eggs” OR “fledgling*” OR “fledging” OR “reproductive success” OR “fitness”)); (2) TS = (“urban*” AND “bird” AND “laying date”); (3) TS = (“urban*” AND “bird” AND “clutch size”); (4) TS = (“urban*” AND “bird” AND “fledglings”). The *list of avian genera* in the first search string consisted of a list of all avian genera and can be found in Supplementary text D (see also acknowledgements). We complemented the search on the Web of Science Core Collection by searching Scopus using search string ‘(1)’ above (Scopus field ‘TITLE‐ABS‐KEY’). Both literature searches, on the Web of Science Core Collection and Scopus, included studies published before the 27th of October 2020. We used the literature search results in these two major search engines to assess the comprehensiveness of our search (see Supplementary Text A for details). These searches found 892, 71, 198, 167 (on the Web of Science Core Collection) and 735 (on Scopus) studies, respectively, which we combined with the studies identified from Chamberlain et al. (2009) and Sepp et al. (2018) to create a list of 2132 (non‐unique) studies (Figure S1). We then de‐duplicated this list using the R package ‘revtools’ (using exact matching of study titles in function ‘*find_duplicates*’, v0.4.1; Westgate, 2019) and by manually inspecting all titles and author lists. Our final list contained 1166 unique studies (Figure S1), which we screened by reading their title and abstract (this first screening step was made by P.C.‐L., C.J.B. and D.M.D.). If the title and/or abstract indicated that the paper could fit our requirements for data collection (see below), we read the study fully, aiming to extract mean, standard deviation (SD) and sample size (*n*) of our life‐history traits of interest for urban and non‐urban bird populations. If SD was not available but authors provided SE, the former was calculated as: SD=SE×√n. Mean and SD were extracted from data quartiles and medians in four effect sizes from two studies following (Luo et al., 2016; Shi et al., 2020). When available, we extracted estimates per breeding season (i.e. papers sometimes reported mean, SD and *n* for urban and non‐urban populations in multiple breeding seasons). If a study reported incomplete information for inclusion in our meta‐analysis (e.g. mean was provided but not SD or SE), we contacted the authors to ask for this missing information (a complete list of authors that provided estimates can be found in the acknowledgements).

### Criteria for inclusion

We were interested in investigating the effects of urbanisation on life‐history traits, with an interest in testing the association between urbanisation and, mean and variation in trait values. Paired urban–non‐urban designs, where an urban population is compared to an adjacent non‐urban population, are a powerful approach to identify the effects of urban living while controlling for temporal and geographical variation, and large‐scale genetic structure among populations (Caizergues et al., 2021; Salmón et al., 2021). Therefore, we included studies if they compared geographically close (i.e. paired) urban and non‐urban populations and reported laying date of the first clutches of the season, clutch size or number of fledglings for the same breeding season across both habitats. When multiple populations were compared along a gradient of urbanisation, we extracted estimates for the two populations at the extremes of the gradient (i.e. most and least urbanised populations). When studies combined estimates across several breeding seasons, we included them in our meta‐analysis if urban and non‐urban populations had been sampled in the same breeding seasons. All effect sizes were extracted by one author (P.C.‐L.). To validate data extraction, another author (M.J.T.) checked 15% of the studies included in the meta‐analysis, comprising 55 effect sizes (17.80% of the final dataset; Supplementary Text B).

Initially, our dataset contained 443 paired urban–non‐urban estimates from 40 bird species and 74 studies. Of these, three observations were removed due to missing sample sizes, 26 observations were removed due to missing SD and 11 observations were removed because their sample size was one (which precludes the calculation of mean and SD). Four observations were removed because they reported a SD of zero (these indeed had very low sample sizes: 3, 2, 7, 2 observations). Our final dataset included 399 comparisons between paired urban–non‐urban populations from 35 bird species and 68 studies (Figure 1; refs.: Antonov & Atanasova, 2003; Bailly et al., 2016; Baldan & Ouyang, 2020; Beck & Heinsohn, 2006; Berardelli et al., 2010; Biard et al., 2017; Boal & Mannan, 1999; Bobek et al., 2018; Brahmia et al., 2013; Caizergues et al., 2018; Capilla‐Lasheras et al., 2017; Cardilini et al., 2013; Charter et al., 2007; Conway et al., 2006; de Satgé et al., 2019; Dhondt et al., 1984; Eden, 1985; Evans & Gawlik, 2020; Gahbauer et al., 2015; Glądalski, Bańbura, Kaliński, Markowski, Skwarska, Wawrzyniak, Zieliński, & Bańbura, 2016; Glądalski, Bańbura, Kaliński, Markowski, Skwarska, Wawrzyniak, Zieliński, Cyżewska, & Bańbura, 2016; Glądalski et al., 2015, 2017, 2018; Gryz & Krauze‐Gryz, 2018; Hajdasz et al., 2019; Hinsley et al., 2008; Ibáñez‐Álamo & Soler, 2010; Isaksson et al., 2008; Isaksson & Andersson, 2007; Jarrett et al., 2020; Kelleher & O'Halloran, 2007; Kettel et al., 2019; Kopij, 2017; Lee et al., 2017; Lin et al., 2015; Liven‐Schulman et al., 2004; Luna et al., 2020; Mcgowan, 2001; Mennechez & Clergeau, 2006; Middleton, 1979; Millsap et al., 2004; Minias, 2016; Morrissey et al., 2014; Newhouse et al., 2008; Partecke et al., 2020; Perlut et al., 2016; Pollock et al., 2017; Preiszner et al., 2017; Rollinson & Jones, 2003; Rosenfield et al., 2019; Schmidt & Steinbach, 1983; Schoech et al., 2007; Schoech & Bowman, 2001; Seress et al., 2012, 2018, 2020; Sharma et al., 2004; Shustack & Rodewald, 2011; Solonen, 2001, 2014; Solonen & Ursin, 2008; Stout et al., 1998; Stracey & Robinson, 2012; Sumasgutner et al., 2014; Thornton et al., 2017; Wawyrzyniak et al., 2015; Welch‐Acosta et al., 2019). Of these 399 comparisons, 151 corresponded to comparisons of laying date (*n* = 32 studies), 119 were comparisons of clutch size (*n* = 42 studies) and 129 were comparisons of number of fledglings (*n* = 48 studies) (Figure S2). Last, there were 363 comparisons for single years (*n* = 47 studies) and an additional 36 comparisons included estimates across multiple years (*n* = 21 studies).

### Meta‐analytic effect sizes

We standardised laying date across studies by coding it as the number of days after the 1st of January (January 1st = 1). Mean laying date estimates across habitats always fell within the same calendar year. For each of the three life‐history traits, we computed the log response ratio (lnRR) and the log coefficient of variation ratio (lnCVR) to investigate differences in mean values and variability between urban and non‐urban populations (Hedges et al., 1999; Nakagawa et al., 2015; Senior et al., 2020). We calculated lnRR and lnCVR along with their associated sampling variances (Nakagawa et al., 2015) using the R function ‘*escalc*’ in the ‘metafor’ R package (v3.4.0; Viechtbauer, 2010). Both lnRR and lnCVR were calculated so that positive values meant higher estimates in urban populations compared to their non‐urban counterparts. Often mean and variance values are positively associated (e.g. Taylor's Law; Cohen & Xu, 2015; Nakagawa & Schielzeth, 2013). Therefore, we chose lnCVR over lnVR (i.e. log total variation ratio; Nakagawa et al., 2015) as the former accounts for the mean–variance relationship (Nakagawa et al., 2015; Senior et al., 2020). However, we carried out sensitivity analysis using, among others, the log total variation ratio (Section ‘Sensitivity analyses’).

### Quantifying habitat heterogeneity and urban index

We calculated habitat heterogeneity from the 3CS LC (Copernicus Climate Change Service Land Cover) and the ESA‐CCI LC (European Space Agency‐Climate Change Initiative Land Cover) land cover products (ESA. Land Cover CCI Product User Guide, 2017; ESA. 3CS Land Cover Product User Guide 2020). These datasets provide methodologically consistent land cover per year and gridded maps from 1992 to 2019, with a global coverage and a spatial resolution of circa 300 m per pixel (0.002778° or 10 arcseconds). Each pixel in the products is classified as one of the 22 land cover categories defined by the UN‐FAO‐LCCS (United Nations Food and Agriculture Organization Land Cover Classification System). From a subset of studies included in our main meta‐analysis, we could extract the coordinates of their urban and non‐urban populations (26 studies out of 68 provided accurate coordinates of their urban and non‐urban populations). Then, we sampled the landscape of these studies by extracting the number of pixels belonging to each land cover category around each urban and non‐urban location (i.e. within a circular buffer around each location). The extraction was performed for several buffer radii from 250 m to 5000 m in intervals of 250 m. Landscape heterogeneity was calculated as the effective number of land covers present in each buffer and computed as the exponential of the Shannon–Wiener diversity index (i.e. Hill's numbers for q=1) (Chao et al., 2014; Hill, 1973), resulting in a measure that not only accounts for the absolute richness of land cover categories but also weights the relative abundance of each category. An urban index was calculated as the proportion of each buffer area categorised as an ‘urban’ land cover type. Land cover data were processed and analysed using R (v.4.2.0; R Core Team, 2022). Geospatial vectorial operations were conducted utilising the ‘sf’ R package (v.1.0–7; Pebesma, 2018) while raster extractions were performed with the ‘raster’ R package (v.3.5–15; Hijmans, 2020). All geospatial analyses were performed in the WSG 1984 projected Coordinate Reference Systems, EPSG: 6326. Additionally, we calculated the distance between each urban and non‐urban pair of populations using the function ‘*pointDistance*’ in the R package ‘raster’. We could retrieve location information for 232 urban versus non‐urban comparisons for laying date, clutch size and number of fledglings, from 11 species and 26 studies between 1992 and 2017 (land cover data were not available before 1992; see above).

### Meta‐analyses

We handled the datasets, ran all analyses and produced visualisations using R (v.4.2.0; R Core Team, 2022). To evaluate the effect of urbanisation on bird life‐history traits, we fitted phylogenetic multilevel (intercept‐only) meta‐analyses for each response term (i.e. lnRR [Model 1] and lnCVR [Model 3]; Table 1) combining the three life‐history traits (i.e. laying date, clutch size and number of fledglings; we also fitted models that separated variation between these traits; see below; Table 1). Both meta‐analytic models estimated four random intercept effects, publication identity (i.e. among‐study variation), population identity (i.e. in several cases, we found multiple studies from the same urban–non‐urban populations pairs), phylogeny (more details below), species identity (i.e. among‐species variation not explained by phylogeny) and an observation ID term. For the intercept‐only models, we estimated total heterogeneity (*I* ^2^) following Nakagawa and Santos (2012) and Senior, Grueber, et al. (2016) as implemented in the R function ‘*i2_ml’* (‘orchaRd’ R package v.0.0.0.9000; Nakagawa et al., 2021).

**TABLE 1:** Description of meta‐models. Model IDs are given sequentially from 1 to 10 to facilitate understanding of methods and results. ‘Data’ refers to whether a given model contained data for all traits of interest (‘All traits’) or models were fitted per trait. Moderator ‘Trait’ is a 3‐level factor with levels ‘Laying date’, ‘Clutch size’ and ‘Number of fledglings’. ‘Equations’ provide references to the Equations described in the methods section, whereas ‘Details’ gives a brief description of each model ID and references to output tables and figures

Model ID	Response	Data	Moderators	Equations	Details
1	lnRR	All traits	Intercept	—	Overall meta‐analysis. Univariate. Table S1. Figure S3
2	lnRR	All traits	Trait	Equation 1	Effect per trait. Trivariate. Tables S2 and S3. Figures 2 and 3, Figure S4
3	lnCVR	All traits	Intercept	—	Overall meta‐analysis. Univariate. Table S1. Figure S5
4	lnCVR	All traits	Trait	Equation 1	Effects per trait. Trivariate. Table 2, Tables S4 and S5. Figures 2 and 3, Figure S4
5	lnCVR	All traits	Trait	Equation 1	Comparison of intra‐annual phenotypic variation. Table 2
6	lnCVR	All traits	Trait	Equations 1, 7 and 8	Comparison of inter‐annual phenotypic. Table 2
7	lnCVR	All traits	Trait + Difference in urbanisation + Difference in habitat heterogeneity	Equation 1 (with additional moderators)	Trivariate. Fitted for different spatial scales. Figure 4
8	SDHM	All traits	Trait	Equation 1	Similar structure as Model 2
9	lnVR	All traits	Trait	Equation 1	Similar structure as Model 4
10	lnSD	Each trait individually	Intercept + Habitat + lnMean	Equation 9	Armed‐based model (Senior, Gosby, et al. 2016)

### Phylogenies

Phylogenetic trees were extracted from the Open Tree of Life (Hinchliff et al., 2015; Rees & Cranston, 2017), using the interface provided by the R package ‘rotl’ (v3.0.12; Michonneau et al., 2016; OpenTreeOfLife et al., 2019). We calculated tree branch length (Grafen, 1989) and generated a phylogenetic correlation matrix to include in all our phylogenetic multilevel meta‐analytic models (Figure 1). We assessed the phylogenetic signal in our meta‐analysis based on the proportion of variation explained by the phylogeny ($I_{\text{phylogeny}}^{2}$; Cinar et al., 2022).

### Modelling heterogeneous variances and correlations among traits

Laying date, clutch size and number of fledglings are often correlated in bird species (Dunn & Møller, 2014; Rowe et al., 1994). To assess whether urbanisation is associated with correlated responses across life‐history traits and to test the robustness of our results to the existence of these correlations, we built trivariate meta‐analytic models of lnRR and lnCVR that allowed us to simultaneously estimate trait‐specific means (i.e. one intercept for each trait—Equation 1), trait‐specific observation ID variances (i.e. one observation ID variance for each trait—Equations 1 and 2) and trait‐specific among‐study variances and correlation among traits (Equations 1 and 3). Including the random‐effects detailed above, our model with heterogeneous variances and among‐study correlations among traits can be written as: (we have omitted the term associated with sampling variance for simplicity—see Nakagawa et al., 2015 for more details)

(1) $$y_{i}=\left[\begin{array}{c} \mu_{\mathrm{LD}}\\ \mu_{\mathrm{CS}}\\ \mu_{\mathrm{NF}} \end{array}\right]+\left[\begin{array}{c} \varepsilon_{i-\mathrm{LD}}\\ \varepsilon_{i-\mathrm{CS}}\\ \varepsilon_{i-\mathrm{NF}} \end{array}\right]+\left[\begin{array}{c} \tau_{t-\mathrm{LD}}\\ \tau_{t-\mathrm{CS}}\\ \tau_{t-\mathrm{NF}} \end{array}\right]+v_{y}+a_{l}+h_{w},$$

(2) $$\begin{array}{c} \varepsilon_{i-\mathrm{LD}}\\ \varepsilon_{i-\mathrm{CS}}\\ \varepsilon_{i-\mathrm{NF}} \end{array}\sim N\left(\left[\begin{array}{c} 0\\ 0\\ 0 \end{array}\right],\left[\begin{array}{c} \sigma_{\varepsilon -\mathrm{LD}}^{2}\\ \sigma_{\varepsilon -\mathrm{CS}}^{2}\\ \sigma_{\varepsilon -\mathrm{NF}}^{2} \end{array}\right]\right),$$

(3) $$\begin{array}{c} \tau_{t-\mathrm{LD}}\\ \tau_{t-\mathrm{CS}}\\ \tau_{t-\mathrm{NF}} \end{array}\sim N\left(\left[\begin{array}{c} 0\\ 0\\ 0 \end{array}\right],\left[\begin{array}{ccc} \sigma_{t-\mathrm{LD}}^{2} & p_{\mathrm{LD}-\mathrm{CS}}\;\sigma_{t-\mathrm{LD}}\;\sigma_{t-\mathrm{CS}} & p_{\mathrm{LD}-\mathrm{NF}}\;\sigma_{t-\mathrm{LD}}\;\sigma_{t-\mathrm{NF}}\\ & \sigma_{t-\mathrm{CS}}^{2} & p_{\mathrm{CS}-\mathrm{NF}}\;\sigma_{t-\mathrm{CS}}\;\sigma_{t-\mathrm{NF}}\\ & & \sigma_{t-\mathrm{NF}}^{2} \end{array}\right]\right),$$

(4) $$v_{y}\sim N\left(0,\sigma_{v}^{2}\right),$$

(5) $$a_{l}\sim N\left(0,{\mathrm{A\sigma}}_{a}^{2}\right),$$

(6) $$h_{w}\sim N\left(0,\sigma_{h}^{2}\right),$$

where $y_{i}$ is the statistic of interest (lnRR or lnCVR) for the *i*th effect size (i=1,2,3,…,k; where k is the number of the effect sizes included in the analysis—that is number of urban–non‐urban paired comparisons). ‘LD’, ‘CS’ and ‘NF’ refer to overall means (μ), variances (σ^2^) and correlations (*ρ*) involving effect sizes for laying date (‘LD’), clutch size (‘CS’) and number of fledglings (‘NF’). $\varepsilon_{i}$ is the observation ID deviation for the *i*th observation, which is assumed to follow a normal distribution with mean zero and variance $\sigma_{\varepsilon -\mathrm{LD}}^{2}$, $\sigma_{\varepsilon -\mathrm{CS}}^{2}$, $\sigma_{\varepsilon -\mathrm{NF}}^{2}$ for laying date, clutch size and number of fledglings respectively. $\tau_{t-\mathrm{LD}}$, $\tau_{t-\mathrm{CS}}$ and $\tau_{t-\mathrm{NF}}$ are the deviations from the mean associated with the *t*th study and trait (‘LD’, ‘CS’ or ‘NF’), each following a multivariate normal distribution with mean of zero and variance–covariance structure detailed in Equation 5 (p provides the correlations between $\tau_{t-\mathrm{LD}}$, $\tau_{t-\mathrm{CS}}$ and $\tau_{t-\mathrm{NF}}$). $v_{y}$ provides the deviation from the overall mean associated with the *y*th population (Equation 4). $a_{l}$ is the phylogenetic effect for the *l*th species, which follows a normal distribution with mean equal to zero and variance–covariance structure given by $\sigma_{a}^{2}$, the variance of the phylogenetic effect, and A, a l by l matrix of distances between species calculated from a phylogenetic tree (Equation 5; details above). $h_{w}$ captures among species variation not explained by the phylogenetic effect and follows a normal distribution around zero and variance $\sigma_{h}^{2}$ (Equation 6).

We compared models with different constraints in the parameters of the variance–covariance structure in Equation 3 to assess the strength of evidence for heterogeneous variances and correlations among traits (see results in Tables S2 and S4). We fitted these trivariate meta‐analytic models in the ‘metafor’ R package (‘*rma.mv*’ function; v3.4.0; Viechtbauer, 2010) using maximum likelihood and compared models using AIC (Akaike Information Criterion; Burnham et al., 2011). We then calculated a ΔAIC value for each model (i.e. the difference in AIC between a given model and the model with the lowest AIC) and used this value to assess the strength of evidence for a given variance–covariance structure. We fitted models with the following constraints in the variance–covariance structure:

- Single variance across traits and zero covariances:
$\sigma_{t-\mathrm{LD}}^{2}=\sigma_{t-\mathrm{CS}}^{2}=\sigma_{t-\mathrm{NF}}^{2}$; and all p=0
- Compound symmetric variance–covariance matrix:
$\sigma_{t-\mathrm{LD}}^{2}=\sigma_{t-\mathrm{CS}}^{2}=\sigma_{t-\mathrm{NF}}^{2}$; and $p_{\mathrm{LD}-\mathrm{CS}}=p_{\mathrm{LD}-\mathrm{NF}}=p_{\mathrm{CS}-\mathrm{NF}}$
- Heteroscedastic compound symmetric variance–covariance matrix:
$\sigma_{t-\mathrm{LD}}^{2},\sigma_{t-\mathrm{CS}}^{2}$ and $\sigma_{t-\mathrm{NF}}^{2}$ can vary freely but $p_{\mathrm{LD}-\mathrm{CS}}=p_{\mathrm{LD}-\mathrm{NF}}=p_{\mathrm{CS}-\mathrm{NF}}$
- Diagonal variance–covariance matrix:
$\sigma_{t-\mathrm{LD}}^{2},\sigma_{t-\mathrm{CS}}^{2}$ and $\sigma_{t-\mathrm{NF}}^{2}$ can vary freely but all p=0
- Unstructured variance–covariance matrix
$\sigma_{t-\mathrm{LD}}^{2},\sigma_{t-\mathrm{CS}}^{2}$, $\sigma_{t-\mathrm{NF}}^{2}$, $p_{\mathrm{LD}-\mathrm{CS}},p_{\mathrm{LD}-\mathrm{NF}}$ and $p_{\mathrm{CS}-\mathrm{NF}}$ can vary freely.

### Within‐ and between‐breeding season differences in phenology and life‐history traits

Urban and non‐urban populations may differ in both within‐ and between‐breeding season variation in life‐history traits. However, differences in variation for these two temporal scales are likely generated by contrasting ecological and evolutionary processes. To disentangle processes operating at these two temporal scales, we performed additional meta‐analyses including (i) urban–non‐urban comparisons within breeding seasons (*k* = 363 comparisons from 47 studies in the original dataset with effect sizes per year; Model 5) and (ii) urban–non‐urban comparisons between breeding seasons (i.e. combining all within‐breeding season estimates from a study; *k* = 36 comparisons present in the original dataset, plus 67 additional comparison calculated from within‐breeding season estimates; see below). When a given study reported estimates across multiple breeding seasons, we calculated between‐breeding season mean and variance as:

(7) $${\bar{x}}_{\text{among}-\text{season}}=\sum_{i=1}^{g}{\bar{x}}_{i}\frac{n_{i}}{N};\text{where},N=\sum_{i=1}^{g}n_{i},$$

(8) $$S_{\text{among}-\text{season}}^{2}=\sum_{i=1}^{g}\frac{n_{i}}{N}s_{i}^{2}+\sum_{i=1}^{g}\frac{n_{i}}{N}\;{\left({\bar{x}}_{i}-{\bar{x}}_{\text{among}-\text{season}}\right)}^{2},$$

where, ${\bar{x}}_{\text{among}-\text{season}}$ and $S_{\text{among}-\text{season}}^{2}$ are mean and variance across multiple breeding seasons. g is the total number of breeding seasons reported by a given study; ${\bar{x}}_{i}$, $s_{i}^{2}$, $n_{i}$, are mean, variance and sample size for each breeding season i. ${\bar{x}}_{\text{among}-\text{season}}$ for a given study is, therefore, the weighted average across breeding seasons (Equation 7); whereas $S_{\text{among}-\text{season}}^{2}$ for a given study is the weighted sum of within‐season variances (first term in Equation 8) and between‐season mean variances (second term in Equation 8).

### Assessing the effect of urbanisation and habitat heterogeneity on differences in phenotypic variation between habitats

We investigated the spatial drivers of differences in phenotypic variation between urban and non‐urban populations using the subset of studies which allowed the quantification of an urban index in urban and non‐urban populations (see above). We first verified that the urban index was indeed higher for urban than for non‐urban populations. We compared the urban index in urban and non‐urban populations at different spatial scales via linear models, with the difference in urban index between population as the response variable and an intercept term. Then, to assess whether the increase in phenotypic variation in urban habitats was predicted by habitat heterogeneity and/or urban index, we ran an additional meta‐regression to explain differences in phenotypic variation between urban and non‐urban populations (i.e. lnCVR), where the difference in habitat heterogeneity and urban index between urban and non‐urban populations were included as continuous moderators. This meta‐regression included 232 urban–non‐urban comparisons from 11 species and 26 studies (i.e. the subset of observations after 1992 for which geolocations were available).

### Sensitivity analyses

We assessed the robustness of our results with several complementary analyses. First, we re‐ran the trivariate lnRR model (Model 2; Table 1) using Hedges' *g* (Hedges, 1981) with heteroscedastic population variances as the response variable (Model 8; Table 1; i.e. ‘SMDH’, calculated using the R function ‘*escalc*’ in the ‘metafor’ R package (v3.4.0; Viechtbauer, 2010)). In addition, we assessed the robustness of the lnCVR results by re‐running the trivariate lnCVR model (Model 4; Table 1) using lnVR as the response variable (i.e. the logarithm of the total variation ration; Nakagawa et al., 2015; Model 9; Table 1). Last, we used an alternative approach that directly models the log of the phenotypic standard deviation (lnSD) to assess differences in phenotypic variation between urban and non‐urban populations (equation 18 in Nakagawa et al., 2015; Model 10; Table 1). We followed the model specification shown in Senior, Gosby, et al. (2016), in short:
(9)

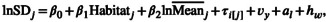

where $\beta_{0}$ is the overall intercept, $\beta_{1}$ is the habitat effect on lnSD (i.e. a $\beta_{1}$ statistically different from zero would indicate that urban and non‐urban populations differ in their phenotypic variation) and $\beta_{2}$ is the slope of the regression of (log) mean values against (log) standard deviations, which is explicitly modelled. $v_{y}$, $a_{l}$ and $h_{w}$ are as per Equation 1. $\tau_{i\left[j\right]}$ is the random effect for the *j*th effect size in the *i*th study. Within each study, effect sizes across habitats are assumed to be correlated; this correlation is calculated by the model (Senior, Gosby, et al., 2016). We applied the model in Equation 9 for each trait independently (i.e. three univariate models, one per trait).

### Publication bias

We assessed the evidence for the existence of two types of publication biases, small‐study and decline effects (time‐lag effects), following Nakagawa et al. (2022). For that, we ran four additional uni‐moderator multilevel meta‐analytic models, two for lnRR and two lnCVR. Each of these models included as a single moderator either the square‐root of the inverse of the effective sample size or the mean‐centred year of study publication (Nakagawa et al., 2022; Trikalinos & Ioannidis, 2005). The variation explained by these moderators (i.e. $R_{\text{marginal}}^{2}$) was calculated using the R function ‘r*2_ml’* (‘orchaRd’ R package v.0.0.0.9000; Nakagawa et al., 2021).

## RESULTS

After systematically inspecting 1166 studies published between 1958 and 2020 (Figure S1), our meta‐analysis included 399 urban–non‐urban comparisons for three bird life‐history traits: laying date (*k* = 151 effect sizes, *n* = 32 studies), clutch size (*k* = 119 effect sizes, *n* = 42 studies) and number of fledglings (*k* = 129 effect sizes, *n* = 48 studies) (Figure 1). This dataset included 35 bird species, with most studies located in the northern hemisphere (Figure 1c).

### Is urbanisation associated with shifts in mean life‐history traits?

**FIGURE 2:**
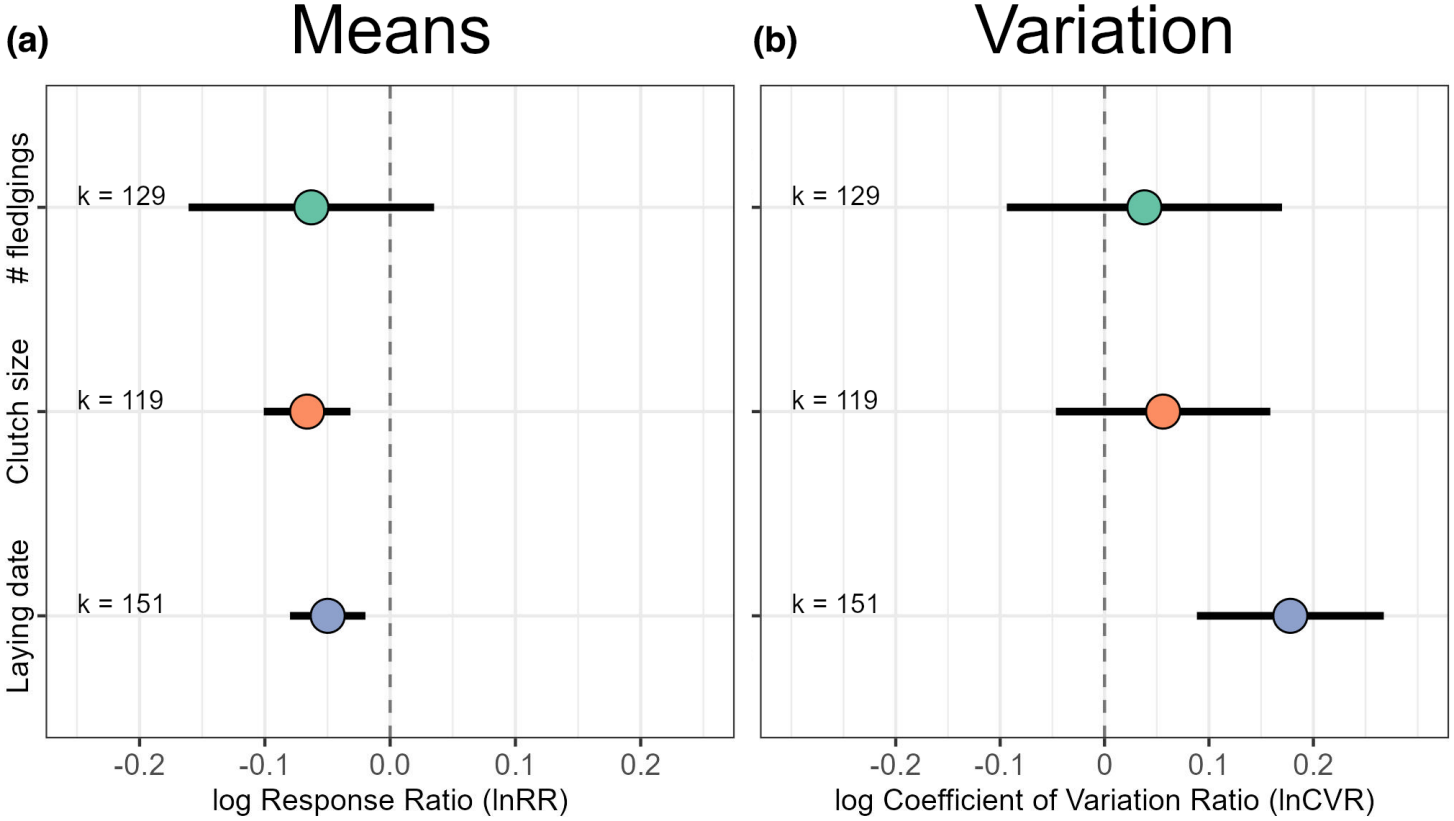
Urban populations have earlier phenology, lower reproductive output and more variable life‐history traits than non‐urban populations. (a) Urban populations laid earlier and had smaller clutches, producing fewer fledglings, than their paired non‐urban populations (illustrated by negative lnRR estimates; Model 2). (b) Our meta‐analysis revealed that variation in life‐history traits was higher in urban populations compared to non‐urban counterparts, with a marked difference between populations in laying date (illustrated by positive estimates of lnCVR; Model 4). Model estimates for (a) lnRR and (b) lnCVR are shown along with their 95% confidence intervals per trait as calculated by our phylogenetic multilevel meta‐analytic models accounting for correlated responses to urbanisation among traits (see Tables S3 and S5 for full model outputs and Figure S3 and S5 for overall meta‐analyses of lnRR and lnCVR). Raw data and model estimates are presented in Figure S4. ‘k’ provides the number of urban–non‐urban comparisons.

**FIGURE 3:**
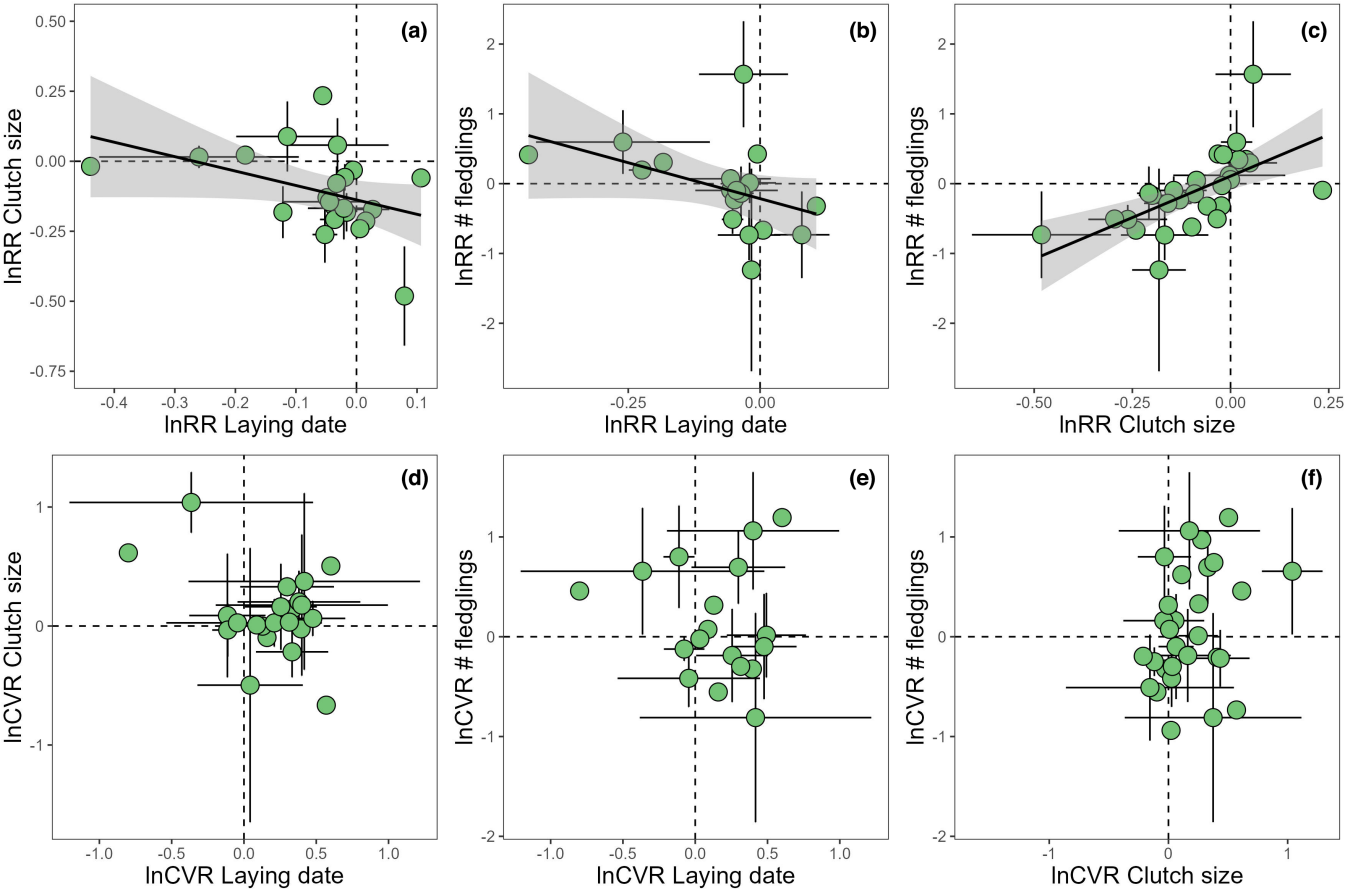
Life‐history traits show a correlated response to urbanisation. Our meta‐analysis investigated correlated responses to urbanisation across the three studied life‐history traits, and revealed strong correlations in log response ratio (lnRR) but not log coefficient of variation ratio (lnCVR). (a) Earlier laying dates in urban populations compared to non‐urban counterparts (i.e. negative values in the x axis) were associated with no differences in clutch size across habitats (i.e. y axis values close to zero), leading to a negative correlation between lnRR for these two traits. (b) A similar pattern was found between lnRR for laying dates and number of fledglings, while (c) lnRR for clutch size and number of fledglings were positively correlated (Tables S2 and S3; Model 2). (d–f) We found no strong statistical evidence for models including correlations across traits in how urbanisation affected phenotypic variation (Tables S4 and S5): (d) differences between habitats in phenotypic variation in laying dates were not associated with differences between habitats in phenotypic variation in clutch size or (e) number of fledglings; and (f) differences between habitats in variation in clutch size were not associated with differences between habitats in variation in number of fledglings. Points represent mean raw values per study ± SE. Regression lines (mean ± SE) in a–c were fitted using linear regressions to illustrate the correlations revealed by our trivariate meta‐analysis (Model 2; Table S3).

We found that urban populations tended to have, on average, 3.6% lower mean values than their non‐urban counterparts, but note that the 95% confidence interval (hereafter ‘CI’) for this estimate overlapped zero (Model 1: lnRR mean estimate [95% CI] = −0.035 [−0.076, 0.005]; Figure S3; Table S1). Total heterogeneity was high ($I_{\text{total}}^{2}$ = 97.8%), with 17.2% of it explained by phylogenetic and species‐specific effects combined ($I_{\text{phylogeny}}^{2}$ = 1.7%; $I_{\text{species}\;\mathrm{ID}}^{2}$ = 15.5%), while 8.4% was explained by differences among studies (Table S1). Further analyses calculating urban effects per trait and accounting for potential covariation in the response to urbanisation across the three focal traits (i.e. using a model with an unstructured variance–covariance matrix; see Methods and Table S2) confirmed that urban populations had indeed lower mean values in every life‐history trait: urban populations laid their eggs earlier (Model 2: lnRR [95% CI] = −0.048 [−0.084, −0.012]; Figure 2a), laid smaller clutches (Model 2: lnRR [95% CI] = −0.066 [−0.107, −0.025]; Figure 2a), and tended to produce fewer fledglings per clutch than non‐urban populations (Model 2: lnRR [95% CI] = −0.070 [−0.171, 0.032]; Figure 2a). This meta‐analytic model estimated different random effect intercepts per trait and allowed for correlations across traits (Model 2; see Methods for details). This model revealed correlations in the response to urbanisation across traits: studies reporting earlier laying date in urban populations also reported more similar clutch size and number of fledglings between populations (i.e. negative correlations between lnRR for laying dates and clutch size; Figure 3a,b). Likewise, studies reporting large differences in clutch size between urban and non‐urban populations also reported large differences between both habitats in number of fledglings (Figure 3c; see ‘Study ID (correlations)’ in Table S3; i.e. correlations among studies in the values of lnRR for each trait).

### Is urbanisation associated with changes in variation in life‐history traits?

The coefficient of phenotypic variation in urban populations was, on average, 4.4% higher than in non‐urban populations, but note that the 95%CI for this estimate overlapped zero (Model 3: lnCVR mean estimate [95% CI] = 0.043 [−0.092, 0.178]; $I_{\text{total}}^{2}$ = 74.3%; Figure S5 and Table S1). 9.1% of the heterogeneity in lnCVR was explained by phylogenetic and species‐specific effects combined ($I_{\text{phylogeny}}^{2}$ = 5.8%; $I_{\text{species}\;\mathrm{ID}}^{2}$ = 3.3%), while differences between studies explained no heterogeneity in lnCVR ($I_{\text{study}\;\mathrm{ID}}^{2}$ = 0.0%; Table S1). A subsequent model of lnCVR separating urban effects on phenotypic variation per trait and accounting for potential covariation across the three investigated traits in the response to urbanisation (see Methods and Table S4) revealed that the overall effect of urbanisation on life‐history trait variation was driven by urban populations having a more variable phenology than their non‐urban counterparts (Model 4: lnCVR mean for laying date [95% CI] = 0.176 [0.084, 0.268], that is 19.2% more variation, on average, in laying date in urban than non‐urban populations). Although the 95%CIs overlapped zero, the direction of the average effects for clutch size and number of fledglings also reflected higher phenotypic variation in urban compared to non‐urban populations (Model 4: lnCVR mean estimates [95% CI]: clutch size = 0.055 [−0.051, 0.160], number of fledglings = 0.037 [−0.096, 0.171]; Figure 2b). We did not find evidence for correlations in lnCVR between the three life‐history traits (Figure 3; the model including correlations among traits scored more than 1.08 AIC points below the top model, which only included independent Study ID random intercepts per trait [Model 4]; Tables S4 and S5).

### What is the temporal and spatial scale at which urbanisation affects phenotypic variation?

Differences in phenotypic variation in laying date between the urban and non‐urban populations arose from differences in variation within breeding seasons (i.e. intra‐annual) rather than between breeding seasons (i.e. inter‐annual; Table 2). While laying dates in urban populations were more variable than in non‐urban populations within breeding seasons (Model 5: lnCVR mean estimate [95% CI] = 0.177 [0.078, 0.281]; Table 2), a subsequent meta‐analytic model isolating effects on phenotypic variation arising from between breeding season fluctuations revealed no difference between urban and non‐urban populations (Model 6: lnCVR intercept mean [95% CI] = 0.074 [−0.014, 0.161]; Table 2). The sample size for this latter meta‐analysis was almost four times smaller than for the meta‐analysis of within breeding season differences in variation; however, the lnCVR estimates were very different between these models: the mean lnCVR within breeding seasons was more than 2.4 times larger than the mean lnCVR among breeding seasons (Table 2).

**TABLE 2:** Differences in variation (lnCVR) in life‐history traits between urban and non‐urban populations at different temporal scales. Urban–non‐urban differences in variation (lnCVR) in laying date, clutch size and number of fledglings per clutch were meta‐analysed to assess differences in variation between urban and non‐urban populations within (‘intra‐annual’) and among (‘inter‐annual’) breeding seasons (e.g. different temporal scales). lnCVR estimates represent meta‐analytic model intercepts following the model structure presented in Table S5; positive values indicate higher variation in urban populations than in non‐urban populations and vice versa. ‘CI’ = confidence interval; ‘*k*’ = sample size. Terms in italic bold highlight lnCVR estimates whose 95%CIs do not overlap zero. See Table 1 for a description of model IDs.

Temporal scale	lnCVR estimate [95% CI]			*k*
	Laying date	Clutch size	Number of fledglings	
Overall [Model 4]	***0.176 [0.084, 0.268]***	0.055 [−0.051, 0.160]	0.037 [−0.096, 0.171]	399
Intra‐annual [Model 5]	***0.177 [0.078, 0.282]***	0.015 [−0.122, 0.152]	0.116 [−0.059, 0.291]	363
Inter‐annual [Model 6]	0.074 [−0.014, 0.161]	0.096 [−0.019, 0.211]	−0.006 [−0.147, 0.135]	103

**FIGURE 4:**
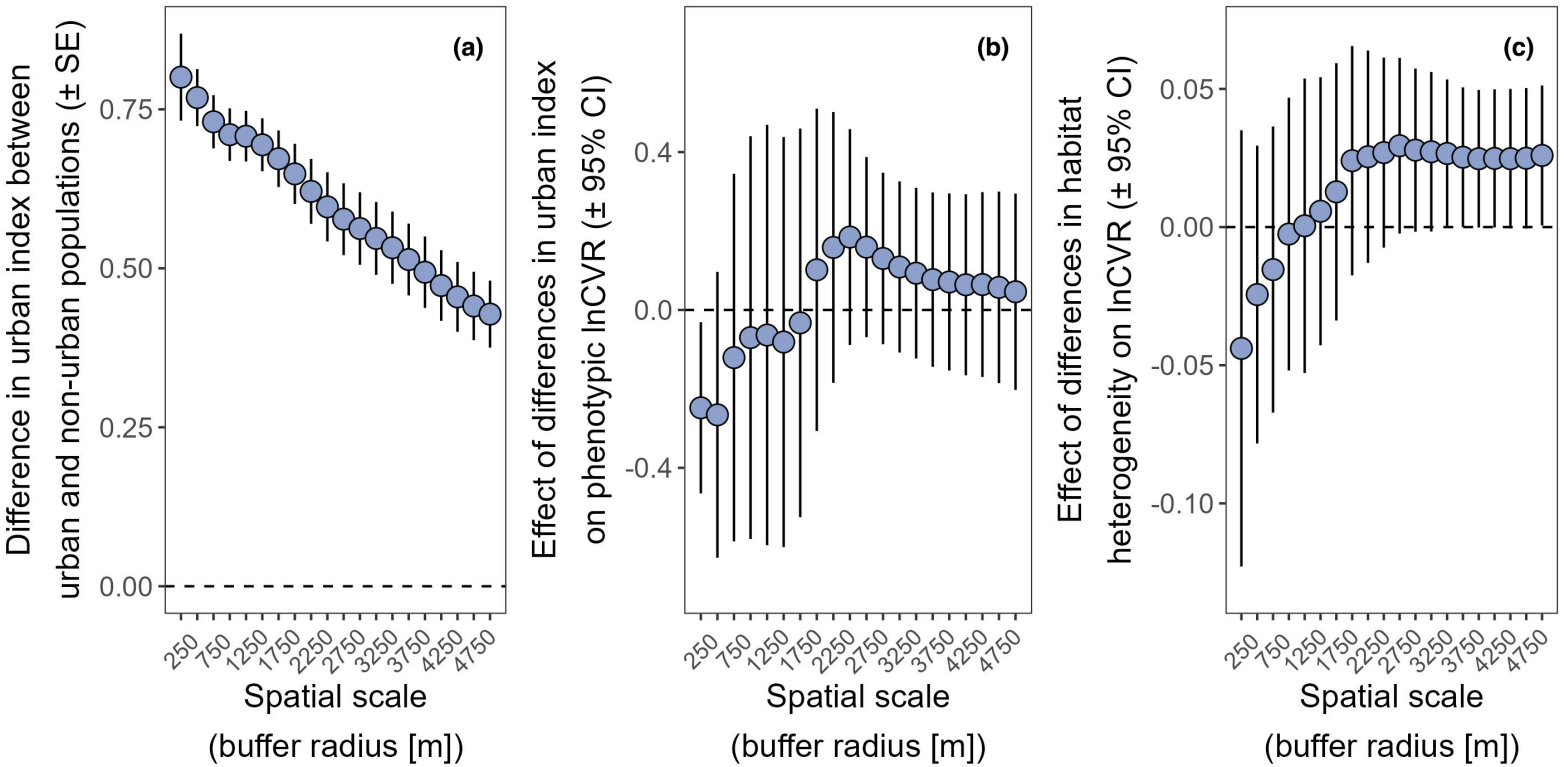
Effects of habitat heterogeneity on the difference in phenotypic variation between urban and non‐urban bird populations (i.e. lnCVR). (a) After quantifying urban index and habitat heterogeneity, we verified that urban populations had higher urban index (i.e. the proportion of landcover at a given spatial scale categorised as ‘urban’ [see methods]). The y axis represents the difference in urban index between urban and non‐urban populations. The positive values observed for all comparisons represent that urban populations had higher urban index than their non‐urban neighbours. (b) Differences in urban index between urban and non‐urban populations did not predict the magnitude of the difference in phenotypic variation between populations (i.e. lnCVR). This figure shows the estimated effect of differences in urban index between populations on lnCVR. Positive values indicate that the higher the difference in urban index between urban and non‐urban populations, the higher the lnCVR value (i.e. larger values of phenotypic variation in urban populations compared to non‐urban counterparts). (c) Differences in habitat heterogeneity between urban and non‐urban populations did positively predict the magnitude of the difference in phenotypic variation between populations (i.e. lnCVR), particularly at large spatial scales. This figure shows the estimated effect of differences in habitat heterogeneity on lnCVR at different spatial scales. Positive values indicate that the higher the difference in habitat heterogeneity between urban and non‐urban populations, the higher the lnCVR value (i.e. larger values of phenotypic variation in urban populations compared to non‐urban counterparts). Points represent mean model estimates ± SE in a, and mean model estimates ±95% confidence intervals (95%CI) in b and c. ‘Spatial scale’ refers to the radius of a circular area centred at each study location and over which urban index and habitat heterogeneity was calculated.

Furthermore, to assess whether urbanisation and/or habitat heterogeneity could explain increased phenotypic variation in urban bird populations, we investigated the extent to which our quantification of urban index and habitat heterogeneity predicted differences in phenotypic variation across populations. First, we confirmed that the urban populations included in our meta‐analysis showed higher levels of urbanisation than paired non‐urban populations regardless of the spatial scale used (urban index in urban population ± SE = 0.669 ± 0.047; urban index in non‐urban population ± SE = 0.021 ± 0.007; at a spatial scale of 2000 m in both cases for reference; Figure 4a). Including the difference in urban index and habitat heterogeneity between paired urban and non‐urban populations as a moderator in a meta‐regression revealed that the more heterogeneous the urban habitat was, the larger the phenotypic variation in this habitat compared to the non‐urban habitat; this effect was particularly strong at medium‐large spatial scales (Figure 4c). Differences in urban index between populations did not strongly explain variation in lnCVR (Figure 4b). Urban and non‐urban populations in each pair were located at a mean distance of 65.4 km (median = 33.1 km; range = [2.4 km, 625.1 km]; *n* = 26 geo‐referenced studies).

### Sensitivity analyses and assessment of publication bias

In line with our main analysis of lnRR (Table S3), using SMDH as the effect size provided negative estimates (i.e. lower phenotypic means in urban populations) for laying dates (SMDH mean estimate [95% CI] = −0.298 [−0.634, 0.039]), clutch size (SMDH mean estimate [95% CI] = −0.145 [−0.420, 0.130]) and number of fledglings (SMDH mean estimate [95% CI] = −0.022 [−0.298, 0.254]) (Model 8 in Table 1). Uncertainty around mean SMDH estimates was high and the 95%CIs overlapped zero. Analysing lnVR instead of lnCVR provided further evidence for increased phenotypic variation in urban populations, particularly for phenology (Model 9 in Table 1): the mean lnVR estimate for laying date was positive and statistically different from zero (lnVR mean estimate for laying date [95% CI] = 0.158 [0.069, 0.247]). As in the lnCVR model, lnVR mean estimates for clutch size and number of fledglings were close to zero (lnVR mean estimate for clutch size [95% CI] = −0.012 [−0.110, 0.056]; lnVR mean estimate for number of fledglings [95% CI] = −0.034 [−0.120, 0.052]). Additionally, the arm‐based model of lnSD for laying date (Model 10 in Table 1) revealed a positive ‘urban’ effect on lnSD: urban populations had lnSD values 0.197 higher than non‐urban populations (i.e. $\beta_{1}$ in Equation 9; 95%CI = [0.122, 0.272]). Laying date (log) mean phenotypic values were positively correlated with lnSD (i.e. $\beta_{2}$ in Equation 9; estimate [95%CI] = 0.416 [0.068, 0.764]). The arm‐based models of clutch size and number of fledglings confirmed correlations between lnMean and lnSD ($\beta_{2}$ in Equation 9 for clutch size, estimate [95%CI] = 0.326 [0.070, 0.582]; for number of fledglings, estimate [95%CI] = 0.231 [0.155, 0.307]), but did not provide evidence for urban effects on phenotypic variation in clutch size or number of fledglings ($\beta_{1}$ in Equation 9 for clutch size, estimate [95%CI] = 0.020 [−0.079, 0.119]; for number of fledglings, estimate [95%CI] = −0.017 [−0.099, 0.065]). We did not find evidence of publication bias in lnRR or lnCVR (Supplementary Text C).

## DISCUSSION

We compiled a global dataset of bird life‐history traits for paired urban and non‐urban populations of the same species to assess how urban living is related to changes in phenotypic means and variation for breeding phenology, reproductive effort and reproductive success. A phylogenetically controlled multilevel meta‐analysis of this dataset confirms a well‐documented effect of urbanisation on mean phenotypes: urban bird populations lay earlier and smaller clutches than their non‐urban counterparts. This model, however, also reveals correlated responses to urbanisation across life‐history traits: for example, the earlier the laying date in urban populations, the smaller the difference in clutch sizes between habitats. Our study goes a step further than previous meta‐analyses in urban ecology by explicitly investigating how urbanisation could impact phenotypic variation. Our findings highlight that urbanisation is associated with both a decrease in mean phenotypes, and an increase in phenotypic variation. Investigating the temporal and spatial scale at which urban phenotypic variation increases revealed hints at the ecological causes and evolutionary consequences.

Urbanisation has been associated with shifts in mean phenotypic values across many organisms (Alberti et al., 2017; Merckx et al., 2018; Santangelo et al., 2022), including birds, which generally show smaller body sizes and lower life‐history trait values in urban habitats (Chamberlain et al., 2009; Sepp et al., 2018; Thompson et al., 2022). Our analyses expand the spatial, temporal and phylogenetic coverage of previous meta‐analyses of the avian literature (Chamberlain et al., 2009; Sepp et al., 2018), and agree on their findings. Our results indicate that urban bird populations lay their eggs earlier and produce smaller clutches, which results in a lower number of surviving nestlings, than their non‐urban neighbouring populations. Note, that our analysis indicates a high total heterogeneity in lnRR ($I_{\text{total}}^{2}$ = 97.8%). This finding indicates large variation (e.g. among studies and species) in how urbanisation associates with changes in mean phenotypes and suggests that additional ecological traits (e.g. diet or migratory strategy) may also affect how populations respond to urbanisation. Our results also indicate that the mean response to urbanisation is correlated among traits. Interestingly, we found that the earlier the laying dates were in urban versus non‐urban populations, the smaller the difference in clutch size and in number of surviving nestlings between habitats. Many bird species show a negative phenotypic and genetic correlation between clutch size and lay date (Dunn & Møller, 2014; Rowe et al., 1994; Sheldon et al., 2003), and these two traits are often hypothesised to co‐evolve (Garant et al., 2008). All else being equal, urban conditions triggering an earlier onset of reproduction (because of e.g. light pollution (Dominoni et al., 2013) or increased resource availability during winter (Schoech et al., 2004)) could indirectly increase clutch size and, therefore, reduce differences in reproductive output between urban and non‐urban populations that arise via other mechanisms (e.g. resource limitation in spring; Seress et al., 2018, 2020). The extent to which mean phenotypic shifts represent adaptive responses to urbanisation in birds, either via genetic changes or plasticity, or are maladaptive, is mostly unknown (Branston et al., 2021; Caizergues et al., 2022; Lambert et al., 2020; Santangelo et al., 2022). Our results, however, highlight that phenotypic shifts in urban populations are widespread and that the response to urbanisation of associated life‐history traits should be investigated together.

Urbanisation has been recently hypothesised to increase phenotypic variation and, indeed, higher variation in morphological traits of urban great tits (*Parus major*) and blue tits (*Cyanistes caeruleus*) has been recently reported (Thompson et al., 2022). Our findings greatly expand the evidence for this emerging hypothesis showing that urbanisation is overall associated with increases in variation in laying date across many bird species. Previous studies have suggested that city characteristics, such as warmer temperatures in early spring due to the urban heat island effect, could allow birds to lay more clutches per season (Schoech et al., 2008; Yeh & Price, 2004), with thereby longer breeding seasons and hence higher phenotypic variation in urban laying dates (a similar result has also been reported in Lepidoptera; Merckx et al., 2021). This effect, however, does not necessarily explain our results as our meta‐analysis only included first clutch laying dates per season. As such, our findings indicate that urban bird populations display more variation in the *onset* of reproduction than their non‐urban neighbours.

Higher phenotypic variation in urban than in non‐urban populations within breeding seasons could be explained by at least two, non‐exclusive, eco‐evolutionary mechanisms: differences in the underlying additive genetic variance in laying date, whereby urban birds have a wider range of breeding values for laying date; and / or differences in habitat heterogeneity influencing plasticity in laying date, whereby urban areas have larger environmental variation than non‐urban habitats (Heisler & Brazel, 2018; Shochat et al., 2006; Strubbe et al., 2020; Thompson et al., 2022). No study to date has investigated whether urban birds show higher additive genetic variance than non‐urban populations. However, genetic analyses of European great tits in urban and non‐urban habitats generally suggest small differences in the magnitude of genetic variation between habitats (Björklund et al., 2010; Caizergues et al., 2021; Salmón et al., 2021). This is, perhaps, not surprising given the high mobility of birds and the fact that gene flow between urban and non‐urban bird populations likely occurs at a large spatial scale (Salmón et al., 2021). Interestingly, some studies have reported weaker selection for laying date in urban areas than in non‐urban habitats, suggesting relaxed selection on phenology in urban birds (Branston et al., 2021; Caizergues et al., 2018), which could increase genetic variation in phenology. Assessing differences in phenotypic variation between urban and non‐urban populations of less mobile species will be important to evaluate how biological traits (e.g. dispersal ability) determine the evolutionary impact of urban ecological conditions. To this end, previous work in mammal and amphibian species that have a lower dispersal ability than birds suggests a similar level of (genetic) variation between urban and non‐urban habitats (Fusco et al., 2021; Richardson et al., 2021).

Habitat complexity differs between urban and non‐urban habitats (Arnfield, 2003; Pickett et al., 2017). Our analyses indicate that differences in urban versus non‐urban habitat heterogeneity could indeed help explain the observed pattern of increased phenotypic variation in urban populations. Several ecological mechanisms could mediate this effect. Urban environments are characterised by an array of microhabitats with varying levels of human pressure, exotic plant species and resource availability. Thus, the intensity and timing of the environmental cues that birds use to time their reproduction could vary at a small local scale, increasing phenotypic variation in phenology in the presence of plasticity. The existence of plastic responses to urban habitat heterogeneity, which our results might indicate, do not preclude selection from acting on urban bird populations. First, plasticity is an important mechanism of adaptation, sometimes aligned in direction with adaptative genetic changes (De Lisle et al., 2022), and indeed is often involved in adaptation to urban environments (Campbell‐Staton et al., 2021; Halfwerk et al., 2019). Second, plastic responses can aid adaptation to urban conditions in the presence of genetic‐by‐environment interactions by increasing genetic variation available for natural selection (Via & Lande, 1985). Addressing which evolutionary mechanisms cause the observed increase in phenotypic variation in urban bird populations is beyond the scope of this study and we acknowledge that these arguments are largely speculative at this point. However, our findings highlight that eco‐evolutionary processes could largely differ between urban and non‐urban bird populations and generate new avenues for future research in urban ecology and evolution.

In agreement with our initial predictions, habitat heterogeneity was associated with the magnitude of the difference in phenotypic variation between urban and non‐urban bird populations. However, we acknowledge that this analysis has several limitations and that the results require cautious interpretation. First, only a subset of published studies provided coordinates for their urban and non‐urban study populations (26 out of 68 published papers). When study site coordinates were provided, only one pair of coordinates per study location was provided, preventing an accurate assessment of the actual area over which a given breeding population was studied. Additionally, it is common in urban eco‐evolutionary studies to monitor several populations within one single city. However, in most studies, spatial information was provided at the scale of the whole city (e.g. a single set of coordinates), preventing the accurate quantification of habitat heterogeneity for every sub‐population within a given urban habitat. These limitations highlight that the ability to perform global meta‐analyses on the effects of urban habitat heterogeneity on phenotypic variation would be greatly improved if individual studies provided accurate coordinates of the location of their study populations. Reporting such information would allow future research synthesis to quantify phenotypic variation within urban populations (e.g. across different sub‐populations in the same city) and between urban and non‐urban populations.

Taken together, our results show that urbanisation is associated with both a decrease in mean phenotypic values and increased phenotypic variation in bird populations. Our analyses also highlight a temporal and spatial mechanism that could generate such differences in phenotypic variation between urban and non‐urban habitats. We show that urban bird populations have a more variable phenology than non‐urban conspecifics within breeding seasons (i.e. differences in phenology across habitats are seemingly not due to between‐year fluctuations) suggesting that the ecological conditions that generate such differences are constant across multiple years. Our coupled spatial analysis indicates habitat heterogeneity and plastic responses as potential eco‐evolutionary drivers generating these results. The eco‐evolutionary implications of higher phenotypic variation in urban environments will likely vary among species (Thompson et al., 2022) and our findings highlight the need for detailed investigation of these consequences. To this end, long‐term studies of individually marked organisms in replicated paired urban and non‐urban environments could be particularly fruitful to unravel whether differences in phenotypic variation between urban and non‐urban populations are caused by differences in underlying genetic variation and/or plastic responses to the urban environment.

## AUTHOR CONTRIBUTIONS

Pablo Capilla‐Lasheras, Megan J. Thompson, Denis Réale, Anne Charmantier and Davide M. Dominoni conceived the study. Pablo Capilla‐Lasheras, Alfredo Sánchez‐Tójar, Claire J. Branston and Davide M. Dominoni performed the literature search. Pablo Capilla‐Lasheras extracted effect sizes from suitable published papers. Megan J. Thompson validated effect size extraction. Pablo Capilla‐Lasheras and Yacob Haddou performed all statistical analysis with advice from Alfredo Sánchez‐Tójar. Pablo Capilla‐Lasheras wrote the first draft of the manuscript with input from Megan J. Thompson, Alfredo Sánchez‐Tójar, Denis Réale, Anne Charmantier and Davide M. Dominoni. All authors read and revised the manuscript.

## FUNDING INFORMATION

P.C.‐L., C.J.B. and D.M.D. were funded by a Highlight Topics grant from the Natural Environment Research Council awarded to D.M.D. (NE/S005773/1).

## CONFLICT OF INTEREST

The authors declare no competing interests.

### PEER REVIEW

The peer review history for this article is available at https://publons.com/publon/10.1111/ele.14099.

### OPEN RESEARCH BADGES

This article has earned Open Data and Open Materials badges. Data and materials are available at https://zenodo.org/record/7010687.

## Supporting information


Appendix S1

## Data Availability

All R scripts and datasets needed to reproduce the analyses presented in this paper are available at https://doi.org/10.5281/zenodo.7010687.
